# The response of a standardized fluid challenge during cardiac surgery on cerebral oxygen saturation measured with near-infrared spectroscopy

**DOI:** 10.1007/s10877-019-00324-w

**Published:** 2019-05-28

**Authors:** Frederik Holmgaard, Simon T. Vistisen, Hanne B. Ravn, Thomas W. L. Scheeren

**Affiliations:** 1 Department of Cardiothoracic Anesthesiology, Heart Centre, Rigshospitalet, University of Copenhagen, Blegdamsvej 9, 2100 Copenhagen, Denmark; 2 Department of Anesthesiology, University Medical Center Groningen, University of Groningen, Groningen, The Netherlands; 3 Department of Clinical Medicine, Aarhus University, Århus, Denmark; 4 Department of Anesthesiology & Intensive Care, Aarhus University Hospital, Århus, Denmark

**Keywords:** Cardiac anaesthesia, Monitoring, Near infrared spectroscopy, Cerebral oximetry, Fluid challenge, Cardiac output

## Abstract

Near infrared spectroscopy (NIRS) has been used to evaluate regional cerebral tissue oxygen saturation (ScO_2_) during the last decades. Perioperative management algorithms advocate to maintain ScO_2_, by maintaining or increasing cardiac output (CO), e.g. with fluid infusion. We hypothesized that ScO_2_ would increase in responders to a standardized fluid challenge (FC) and that the relative changes in CO and ScO_2_ would correlate. This study is a retrospective substudy of the FLuid Responsiveness Prediction Using Extra Systoles (FLEX) trial. In the FLEX trial, patients were administered two standardized FCs (5 mL/kg ideal body weight each) during cardiac surgery. NIRS monitoring was used during the intraoperative period and CO was monitored continuously. Patients were considered responders if stroke volume increased more than 10% following FC. Datasets from 29 non-responders and 27 responders to FC were available for analysis. Relative changes of ScO_2_ did not change significantly in non-responders (mean difference − 0.3% ± 2.3%, p = 0.534) or in fluid responders (mean difference 1.6% ± 4.6%, p = 0.088). Relative changes in CO and ScO_2_ correlated significantly, p = 0.027. Increasing CO by fluid did not change cerebral oxygenation. Despite this, relative changes in CO correlated to relative changes in ScO_2_. However, the clinical impact of the present observations is unclear, and the results must be interpreted with caution.

**Trial registration:**http://ClinicalTrial.gov identifier for main study (FLuid Responsiveness Prediction Using Extra Systoles—FLEX): NCT03002129.

**Electronic supplementary material:**

The online version of this article (10.1007/s10877-019-00324-w) contains supplementary material, which is available to authorized users.

## Introduction

In the last decades, near infrared spectroscopy (NIRS) monitoring has gained interest as a tool to monitor cerebral oxygenation and perfusion during cardiac surgery in an attempt to minimize cerebral complications [1–4]. NIRS works by measuring cerebral tissue oxygen saturation (ScO_2_) and reflects an approximately 25/75 arterial/venous saturation ratio, depending on the device used [5, 6]. Numerous intervention algorithms to mitigate and reverse cerebral desaturation have been published, of which the one published in 2007 appears widely adopted [7]. Application of this hierarchical algorithm has lowered the time with cerebral desaturation measured by NIRS [8–11]. However, it is not clear which part(s) of the algorithm is the successful one to convert an ongoing desaturation. Part of the intervention algorithm is to increase cardiac output (CO) if cerebral desaturation occurs as indicated by decreased ScO_2_. However, this intervention per se has been tested primarily in non-cardiac surgery with diverging findings [12–15].

The aim of this study was to elucidate the difference in ScO_2_ after versus before a standardized 5 mL/kg ideal body weight fluid challenge (FC). Furthermore, we studied the association between relative changes in ScO_2_ and CO during a standardized FC in hemodynamic responders and non-responders to a FC in adult patients undergoing cardiac surgery. Responders were defined as patients with an increase in stroke volume, SV > 10% following FC.

We hypothesized that in responders ScO_2_ would increase, as opposed to non-responders. Furthermore, we hypothesized that relative changes of CO and ScO_2_ would correlate.

## Methods

### Study setting

This study is a retrospective substudy of the FLuid Responsiveness Prediction Using Extra Systoles (FLEX) trial [16]. The trial was conducted at the University Medical Center Groningen (UMCG), The Netherlands between January 2017 and June 2017. The FLEX study was approved by the local Institutional Review Board (METc UMCG number 2016.449, ABR number NL58966.042.16) and registered at http://ClinicalTrial.gov (NCT03002129).

### Participants

All participants in the FLEX trial were older than 18 years of age and scheduled for elective coronary artery bypass grafting with no additional procedures, with or without the use of cardiopulmonary bypass (CPB). Exclusion criteria were preoperative left ventricular ejection fraction < 35%, kidney function requiring haemodialysis, and heart rhythm disturbances such as atrial fibrillation or frequent extra systoles. Written informed consent was obtained from all patients included.

### Study protocol

The study protocol and the primary results from the FLEX trial have been previously published [16]. In short, all patients received a standardized FC (5 mL/kg ideal body weight of lactated Ringer; Baxter, Utrecht, The Netherlands) at two time points during surgery. FC1: after induction of anaesthesia and placement of the central venous catheter and before surgical incision. FC2: during preparation of the left internal mammarian artery. Changes to all other infusion rates as well as vasoactive interventions were avoided during the infusion periods, which were approximately 5 min.

### Data acquisition

#### Cerebral oximetry

NIRS monitoring was obtained with self-adhesive sensors (Medtronic/Covidien INVOS Cerebral/Somatic Oximetry Adult Sensors—Medtronic, Minneapolis, USA) placed bilaterally on the patient’s forehead before induction of anaesthesia. The sensors were connected to a Covidien/Medtronic INVOS 5100c Cerebral/Somatic Oximeter monitor (Medtronic, Minneapolis, USA). Data was recorded in the electronic hospital patient data management system developed to sample data during cardiac surgery (CAROLA, RIVM Centrum Extreme Veiligheid, Bilthoven, The Netherlands) with ScO_2_ baseline marked before anaesthesia related preoxygenation and sampled every 30 s and no in-unit data storage was used. Data was exported to Excel format after surgery.

All variables analysed were mean values of left and right channel. In case a patient had only unilateral NIRS readings one channel was used for analysis.

#### Hemodynamic measurements and alignment to the NIRS readings

All patients were equipped with FloTrac sensors, which were connected to the EV1000 hemodynamic monitor (both Edwards Lifesciences, Irvine, USA) for continuous measurement of SV, CO, and mean arterial pressure (MAP). The EV1000 monitor sampled data every 20 s and all data was later exported to Excel format. FC was marked in the monitor system. MAP was also recorded by the CAROLA system and therefore MAP time series were used to align data for CO from the EV1000 monitor and ScO_2_ values from the CAROLA system. All values were analysed from the last registered value before FC start and then for the following period of the FC in 1-min intervals. Last extracted value was the first value registered after FC was ended.

Not all patients had complete NIRS readings and hemodynamic data, since the use of NIRS was dependent on the preference of the anaesthetist. To maximize the output from the available data FC1 and FC2 were pooled for analysis.

Haematocrit levels from arterial blood gasses were extracted from the CAROLA system as the first and last value accessible in the procedure.

### Outcome

#### Regional cerebral oximetry

The primary outcome was to evaluate relative changes in ScO_2_ during two FCs. Furthermore, the absolute difference in ScO_2_ was evaluated for each individual as well as the correlation of ScO_2_ and CO.

### Statistical analysis

Statistical analyses were performed using SPSS (IBM Corp. Released 2013. IBM SPSS Statistics for Windows, Version 22.0. Armonk, NY: IBM Corp.).

All analyses were conducted for the whole sample of datasets and subsequently stratified for non-responders and fluid responders, except for correlation analysis which was conducted for the whole sample only.

The normality of data distribution was evaluated by visual inspection of quantile–quantile plots. Normally distributed data are presented as mean ± standard deviation (SD), otherwise as median and interquartile range (IQR). Normally distributed data were compared with paired sample *t* test for difference between different time points. Student’s t-test was used to test for difference between groups. Categorical data are presented as numbers and percentages and compared with Pearson’s Chi square test or Fisher’s exact test. Statistical significance was assessed at the 5% level.

Correlation was tested with the Pearson correlation coefficient.

No sample size calculation was performed since this study was a secondary analysis of an already finished trial. Statistical power is expressed through the reported confidence intervals.

## Results

Sixty-one patients were included in the FLEX study. Twenty-seven patients had complete sets of hemodynamic data and NIRS data at FC1. At FC2, 29 patients had complete datasets. In total, this allowed analysis of 56 complete datasets comprising 29 non-responders and 27 fluid responders datasets from 31 unique patients.

Preoperative characteristics, medication, comorbidity and intraoperative data are presented in Table 1 for patients with complete datasets at both FC1 and FC2 (n = 25). Tables 1A and 1B (Appendix S1) illustrate that there was no difference in any of the pre-operative variables for non-responders vs. fluid responders at either FC1 or FC2.

**Table 1:** Patient characteristics

Patients with complete data at both FC1 + FC2 (n = 25)	
Preoperative characteristics	
Age	67.2 ± 10.6
BMI	28.2 ± 3.9
Male gender	22 (88%)
Medication	
Beta blocker	19 (76%)
Calcium channel blocker	7 (28%)
ACE inhibitor	17 (68%)
Diuretics	3 (12%)
Statins	22 (88%)
Comorbidity	
ASA physical score	3.0 ± 0.2
Diabetes	6 (24%)
COPD	5 (20%)
Hypercholesterolemia	14 (56%)
Hypertension	19 (64%)
Intraoperative data	
Infused fluid at FC (mL)	380 ± 50
Hct start procedure (%)	38 ± 4
Hct end procedure (%)	33 ± 5
Hct difference start–end (%)	5 ± 3
OPCABG (opposite to on pump)	23 (92%)

Values are presented as means with ± standard deviation and frequency with (percentage)

*FC* fluid challenge, *BMI* body mass index, *ACE* angiotensin-converting-enzyme, *ASA* American Society of Anaesthesiologist classification of physical health, *COPD* chronic obstructive pulmonary disease, *Hct* haematocrit, *OPCABG* off-pump coronary artery bypass grafting

Table 2 illustrates the difference in absolute values and relative changes for CO and ScO_2_ before and after FC. In general, CO and MAP increased significantly for both non-responders and responders. CO before FC for fluid responders were markedly lower than the corresponding CO for non-responders (3.3 ± 0.8 L/min vs. 4.5 ± 1.4 L/min, p < 0.001).

**Table 2:** Analysed variables before fluid challenge and immediately after fluid challenge

	Before	After	Mean difference	95% CI	p
All patients: FC1 + FC2. 56 datasets					
ScO_2_ (%)	66 ± 6	66 ± 6	0 ± 2	− 0.2; 1.0	0.234
ScO_2_ rel. (%)	100^a^	100.6 ± 3.7	0.6 ± 3.7	− 0.4; 1.6	0.217
CO (L/min)	3.9 ± 1.3	4.4 ± 1.3	0.5 ± 0.5	0.3; 0.6	< 0.001
CO rel. (%)	100^a^	112.7 ± 15.3	12.7 ± 15.3	8.3; 15.9	< 0.001
CI (L/min/m^2^)	2.0 ± 0.6	2.2 ± 0.6	0.2 ± 0.2	0.1; 0.3	< 0.001
SV (mL)	75 ± 23	84 ± 23	9 ± 8.5	7; 11	< 0.001
SVI (mL/m^2^)	38 ± 11	42 ± 11	5 ± 4	3; 6	< 0.001
MAP (mmHg)	73 ± 13	77 ± 13	4 ± 7	2; 6	< 0.001
HR (bpm)	52 ± 8	51 ± 8	− 1 ± 3	0.0; − 1.5	0.049
Non-responders: FC1 + FC2. 29 datasets					
ScO_2_ (%)	66 ± 6	66 ± 7	0 ± 2	− 0.7; 0.4	0.555
ScO_2_ rel. (%)	100^a^	99.7 ± 2.3	− 0.3 ± 2.3	− 1.2; 0.6	0.534
CO (L/min)	4.5 ± 1.43	4.7 ± 1.5	0.2 ± 0.4	0.0; 0.3	0.015
CO rel. (%)	100^a^	104.1 ± 10.5	4.1 ± 10.5	0.1; 8.1	0.044
CI (L/min/m^2^)	2.3 ± 0.6	2.4 ± 0.7	0.1 ± 0.2	0.1; 0.2	0.012
SV (mL)	84 ± 26	88 ± 28	4 ± 6	1; 6	0.002
SVI (mL/m^2^)	43 ± 11	45 ± 12	2 ± 4	1; 3	0.004
MAP (mmHg)	76 ± 12	79 ± 14	3 ± 6	1; 5	0.016
HR (bpm)	54 ± 9	53 ± 9	1 ± 3	− 1; 1	0.588
Responders: FC1 + FC2. 27 datasets					
ScO_2_ (%)	66 ± 7	67 ± 6	1 ± 3	− 0.1; 2.0	0.084
ScO_2_ rel. (%)	100^a^	101.6 ± 4.6	1.6 ± 4.6	− 0.3; 3.4	0.088
CO (L/min)	3.3 ± 0.8	4.0 ± 1.0	0.7 ± 0.4	0.5; 0.9	< 0.001
CO rel. (%)	100^a^	122 ± 14.2	22.0 ± 14.0	16.0; 28.0	< 0.001
CI (L/min/m^2^)	1.6 ± 0.4	2.0 ± 0.5	0.4 ± 0.2	0.2; 0.4	< 0.001
SV (mL)	66 ± 15	80 ± 17	15 ± 7	12; 18	< 0.001
SVI (mL/m^2^)	32 ± 8	39 ± 9	7 ± 3	6; 9	< 0.001
MAP (mmHg)	75 ± 13	80 ± 13	5 ± 8	2; 8	0.005
HR (bpm)	51 ± 8	50 ± 8	1 ± 3	0; 2	0.027

Values are presented as means with ± standard deviation

*FC* fluid challenge, *CO* cardiac output, *CI* cardiac index, *SV* stroke volume, *SVI* stroke volume index, *MAP* mean arterial pressure, *HR* heart rate, *ScO*_*2*_ cerebral oxygen saturation, *bpm* beats per minute

^a^Index value: before FC = index 100

The differences in relative changes in ScO_2_ for fluid responders (mean difference 1.6% and 95% CI − 0.3; 3.4, p = 0.088) and non-responders (mean difference − 0.3% and 95% CI − 1.2; 0.6, p = 0.534) was not significant. The ScO_2_ difference in absolute values before and after FC was not significant for either fluid responders (66 ± 7% vs. 67 ± 6%, p = 0.084) or non-responders (66 ± 6% vs. 66 ± 7%, p = 0.555).

**Fig. 1:**
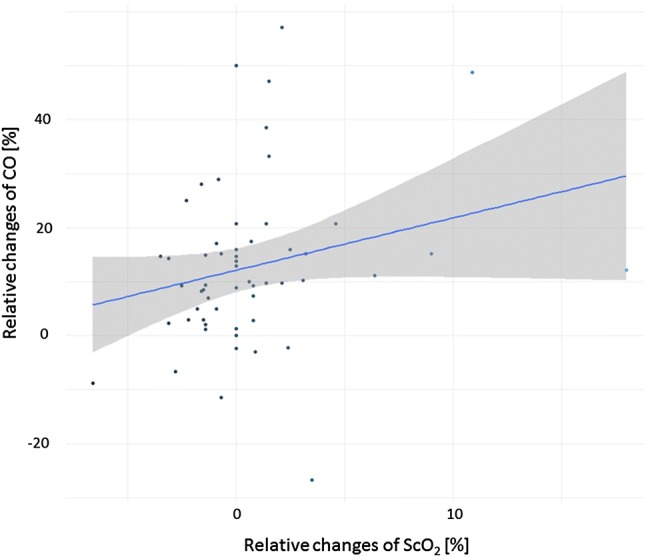
Scatterplot illustrating ScO_2_ and CO at the end of fluid challenge expressed as the relative change to the value before fluid challenge. Illustrated with trendline and confidence interval

**Fig. 2:**
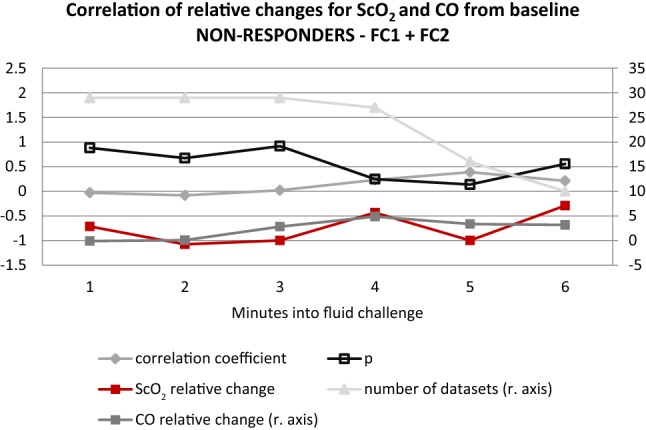
Graph illustrating the relative changes and the correlation between ScO_2_ and CO minute by minute into the fluid challenge for fluid challenge non-responders

**Fig. 3:**
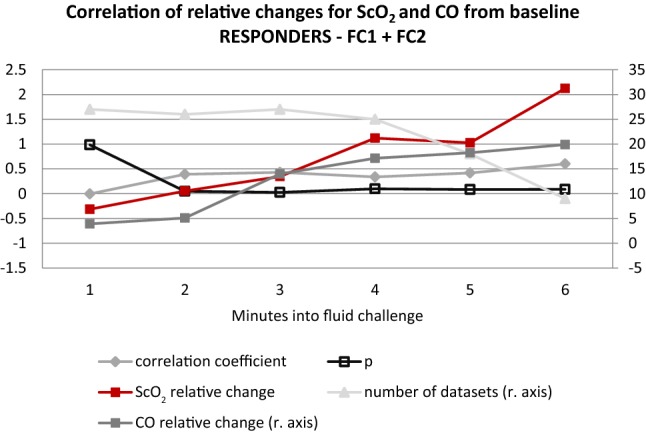
Graph illustrating the relative changes and the correlation between ScO_2_ and CO minute by minute into the fluid challenge for fluid challenge responders

CO and ScO_2_ obtained at the end of FC as relative change to the value before FC are plotted in Fig. 1 and the correlation coefficient was 0.295, p = 0.027. In Fig. 2 (fluid non-responders) and Fig. 3 (fluid responders) the relative changes minute by minute during the FC are presented, showing that the correlations are driven by the fluid responders.

## Discussion

The main finding of the present study was that the ScO_2_ did not change for both responders and non-responders of a FC during cardiac surgery. Despite this, relative changes of CO and ScO_2_ correlated significantly.

It is complicated to compare the results of the present study to the existing literature head-to-head, due to heterogeneity in study designs and settings. Our study is methodologically different from many other studies investigating the hemodynamic effects of a FC, since we had pre-specified time points for the FCs, which we integrated with standard clinical care of our patients (i.e. those accommodating the inclusion criteria). Across fluid responsiveness studies, around 50% of included patients are non-responders to a FC [17]. This is similar in our study, despite the difference in study design. While a different design could have altered the study findings, we find it difficult to speculate what differences to expect.

In the most frequently used intervention algorithm [7] the suggestion to increase ScO_2_ through an increase in CO is based on two small studies: one study reporting that ScO_2_ decreased in patients with normotensive acute heart failure and improved when heart failure was treated [12], and one study showing that ScO_2_ decreased during exercise in patients with left ventricular dysfunction [13]. In the CPB setting it has previously been described in a study testing the intervention algorithm, that increasing pump blood flow was the most successful instrument to minimize cerebral desaturation measured with NIRS [8] even though different pump flow levels have been shown not to affect the cerebral blood flow during CPB [18]. Furthermore, a recently published physiological proof of concept study showed that an increase in CPB pump flow lead to an increase in MAP and an increase in ScO_2_ whereas administration of phenylephrine and vasopressin increased MAP but decreased ScO_2_ [19]. In a randomised trial with two distinct levels of MAP during CPB with fixed pump flow the high MAP target group had lower NIRS values compared to the low MAP target group [20]. Conversely, in the off-pump setting, a study investigating the relationship of central venous oxygen saturation and ScO_2_ during a FC after cardiac surgery found no differences in ScO_2_ before and after FC for either fluid responders or non-responders [14]. However, central venous oxygen saturation was significantly higher for responders after FC whereas it did not change in non-responders. Unfortunately, we did not measure central venous oxygen saturation in the present study at relevant time points.

It was previously described that even though the patient remains within the MAP limits of cerebral autoregulation, the changes in CO can affect ScO_2_ [21]. The MAP levels for the patients in the present study also stayed within the assumed limits of cerebral autoregulation, as presented in Table 2, both before and after FC for both fluid responders and non-responders, although it has been shown that the limits of autoregulation may differ markedly between individuals [22–24]. With regard to cerebral autoregulation it was previously described that the lower limit of autoregulation seems to vary when the central blood volume or CO were lowered [25–28], which needs to be taken into account when evaluating the effect of a FC. In cases where CO is distinctively low, a FC may generate more pronounced responses in cerebral blood flow and subsequently in ScO_2_. Another factor to keep in mind when interpreting the effect of a FC on ScO_2_ is the possible “contamination” of the signal by extracranial perfusion [29], potentially causing a false increase in the NIRS readings. No matter the underlying explanation, we believe the resulting effect of a FC on ScO_2_ can be evaluated per se, which is further emphasized by the Figs. 2 and 3 illustrating an immediate response minute by minute during the FC. We chose to report both relative and absolute values of ScO_2_ since baseline values can vary markedly between individuals [30]. To facilitate the clinical interpretation, we kept relative changes as the primary outcome since it is an optimal way to reflect results in the individual patient due to individual differences in baseline values.

The relative increase in ScO_2_ was on average 2% for fluid responders, in absolute ScO_2_ values this increase was 1%, but both turned out statistically non-significant.

Despite the statistically significant observations of correlation between CO and ScO_2_, one must keep in mind that the clinical relevance of a difference as found in the present study is uncertain. It is very difficult to define a clinically relevant threshold of ScO_2_ levels and the definition of cerebral desaturation assessed with NIRS is vague [31]. Patient characteristics, or intraoperative data including haematocrit, can possibly influence the ScO_2_ readings [32, 33]. We observed a comparable haemodilution during surgery for fluid responders and non-responders—obviously due to the administered fluid in each FC. Therefore, when interpreting the results of the present study, the effect on ScO_2_ of a FC may be less than expected, since the tool to increase CO in this study also generates haemodilution. Therefore, other methods to increase CO without causing haemodilution (e.g. blood transfusions) might show a more pronounced increase in ScO_2_.

The present study has several limitations. Since this study is a substudy and a retrospective analysis of a clinical trial, no sample size calculation was performed. The main result was not significant and it may be caused by a type 2 error. As this study is a retrospective analysis, it was not designed to test the effect of a FC with a higher volume, which may have caused a more distinct response in ScO_2_, since hemodynamic variables demonstrated signs of hypovolaemia before—and for some patients after—FC. Only half of the possible data sets were complete and suitable for analysis and therefore no imputation method was used. The NIRS readings can possibly differ between different manufactures, as previously reported [29]. Therefore, the results may be interpreted with caution when comparing it with that of other studies using different NIRS devices.

In conclusion, the findings of the present study support the current guidelines to increase CO when it comes to maintain ScO_2_ values, but only in conditions where patients are fluid responsive. The clinical impact of small deviations in ScO_2_ on patient outcome is barely described and this study is only indicative that ScO_2_ may be augmented through fluid-induced increases in CO due to the demonstrated correlation between relative changes in ScO_2_ and CO.

## Electronic supplementary material

Below is the link to the electronic supplementary material.

Supplementary material 1 (PDF 347 kb)

## Data Availability

The dataset used and analyzed in the present study is available from the corresponding author on request.
