# Supplementary material for: The response of a standardized fluid challenge during cardiac surgery on cerebral oxygen saturation measured with near-infrared spectroscopy
Source: J Clin Monit Comput. 2019 May 28;34(2):245–51. doi: 10.1007/s10877-019-00324-w (PMC7080680; doi:10.1007/s10877-019-00324-w)
Supplement: Supplementary file 1 — Supplementary material 1 (PDF 347 kb) [file 10877_2019_324_MOESM1_ESM.pdf]

**Patients with complete data at both FC1 + FC2 (n = 25)** ***stratified on non-responders vs. responders at FC1***			
	**Non-responders**	**Responders**	**p**
Preoperative characteristics			
Age	67.55 ± 11.22	67.06 ± 10.62	0.914
BMI	27.77 ± 4.12	28.39 ± 3.83	0.712
Male gender	7 (77.8%)	15 (93.8%)	0.530
Medication			
Beta blocker	8 (88.9%)	11 (68.8%)	0.364
Calcium channel blocker	2 (22.2%)	5 (31.3%)	1.000
ACE inhibitor	5 (55.6%)	12 (75%)	0.394
Diuretic	0 (0%)	3 (18.8%)	0.280
Statins	8 (88.9%)	14 (87.5%)	1.000
Comorbidity			
ASA score	3.0 ± 0	2.94 ± 0.25	0.465
Diabetes	2 (22.2%)	4 (25%)	1.000
COPD	2 (22.2%)	3 (18.8%)	1.000
Hypercholesterolemia	4 (44.4%)	10 (62.5%)	0.434
Hypertension	5 (55.6%)	11 (68.8%)	0.671
Intraoperative data			
Infused fluid at FC [mL]	364.44 ± 46.87	389.38 ± 50.95	0.240
Hct start procedure [%]	36.73 ± 3.88	38.75 ± 3.84	0.346
Hct end procedure [%]	32.1 ± 6.52	33.98 ± 4.49	0.472
Hct difference start-end [%]	4.63 ± 2.7	4.8 ± 2.94	0.915
OPCAB (opposite to on pump)	9 (100%)	14 (87.5%)	0.520

Table 1A

**Patients with complete data at both FC1 + FC2 (n = 25)** ***stratified on non-responders vs. responders at FC2***			
	**Non-responders**	**Responders**	**p**
Preoperative characteristics			
Age	67.0 ± 9.47	73.6 ± 9.24	0.011
BMI	28.18 ± 3.41	28.15 ± 4.66	0.987
Male gender	13 (86.7%)	9 (90%)	1.000
Medication			
Beta blocker	11 (73.3%)	8 (80%)	1.000
Calcium channel blocker	5 (33.3%)	2 (20%)	0.659
ACE inhibitor	11 (73.3%)	6 (60%)	0.667
Diuretic	1 (6.7%)	2 (20%)	0.543
Statins	13 (86.7%)	9 (9%)	1.000
Comorbidity			
ASA score	2.93 ± 0.26	3.0 ± 0	0.426
Diabetes	4 (26.7%)	2 (20%)	1.000
COPD	4 (26.7%)	1 (10%)	0.615
Hypercholesterolemia	10 (66.7%)	4 (40%)	0.241
Hypertension	11 (73.3%)	5 (50%)	0.397
Intraoperative data			
Infused fluid at FC [mL]	374.33 ± 55.22	389.5 ± 42.19	0.470
Hct start procedure [%]	38.63 ± 3.52	37.52 ± 4.84	0.585
Hct end procedure [%]	33.78 ± 3.58	32.94 ± 6.61	0.742
Hct difference start-end [%]	4.91 ± 3.34	4.58 ± 2.36	0.813
OPCAB (opposite to on pump)	14 (93.3%)	9 (90%)	1.000

Table 1B

Journal of clinical monitoring and computing
The response of a standardized fluid challenge during cardiac surgery on cerebral oxygen saturation measured with near-infrared spectroscopy
Corr. Author: Frederik Holmgaard
